# PROBIOTICS AND NUTRIENT SUPPLEMENTS IN MILD COGNITIVE IMPAIRMENT: A SYSTEMATIC REVIEW AND META-ANALYSIS

**DOI:** 10.1093/geroni/igae098.4055

**Published:** 2024-12-31

**Authors:** Mohamed Elhassan, Linda Ahmed, Rawaa Adil, Leena Mohamed, Ahmed Mussaad, Nadir Abdelrahman

**Affiliations:** Royal College of Surgeons Ireland, Waterford, Dublin, Ireland; Tele Geriatric Research Fellowship, Tele Geriatric Research Fellowship, Michigan, United States; Tele Geriatric Research Fellowship, Tele Geriatric Research Fellowship, Michigan, United States; Tele Geriatric Research Fellowship, Tele Geriatric Research Fellowship, Michigan, United States; Tele Geriatric Research Fellowship, Tele Geriatric Research Fellowship, Michigan, United States; Michigan State University, East Lansing, Michigan, United States

## Abstract

Mild cognitive impairment (MCI) is a syndrome marked by cognitive decline beyond normal expectations for age and education, without significantly disrupting daily activities. MCI is a recognized risk factor for dementia, and early detection may allow for secondary prevention. This study evaluates the impact of nutrient supplements and probiotics on MCI patients. Databases including PubMed, Cochrane, Google Scholar, and Science Direct were searched for studies published between 2019 and 2024. Randomized controlled trials (RCTs) involving MCI patients treated with nutrient supplements or probiotics, with cognitive function assessed via the MMSE, were included. Studies with dementia patients were excluded. Data extraction was done independently by two reviewers. A random-effects meta-analysis was conducted to pool standardized mean differences (SMDs). Seven RCTs, totaling 523 participants, were included. Both probiotics and nutrient supplements significantly improved cognitive function, as measured by MMSE, compared to controls (SMD = 0.77, 95% CI: 0.33 to 1.21). Subgroup analysis revealed that nutrient supplements significantly enhanced MMSE scores compared to controls (SMD = 0.8, 95% CI: 0.46 to 1.14). MCI remains a complex issue in geriatric medicine due to ongoing debates over diagnosis and treatment. Currently, no FDA-approved medication exists for MCI. Most studies and guidelines advise against using Dementia drugs like Memantine and Donepezil for MCI due to limited efficacy and potential side effects. However, promising results from recent trials on nutrient supplements and probiotics suggest these could be integrated into future treatment protocols.

